# Tooth morbidity and its impact on oral related quality of life in elderly tribal population-The Irula experience

**DOI:** 10.1186/s12903-025-05628-9

**Published:** 2025-02-18

**Authors:** Margret Beaula Alocious Sukumar, Roshni Mary Peter, Alex Joseph

**Affiliations:** 1 Division of Epidemiology, SRM School of Public Health, SRM Institute of Science and Technology, Kattankulathur, Chennai, Tamil Nadu India; 2 Department of Community Medicine, SRM Medical College Hospital and Research Centre, SRM Institute of Science and Technology, Kattankulathur, Chennai, Tamil Nadu India; 3 Division of Epidemiology, SRM School of Public Health, SRM Institute of Science and Technology, Kattankulathur, Chennai, Tamil Nadu India

**Keywords:** Oral, Quality of life, Tribes, Elderly, Tooth morbidity

## Abstract

**Introduction:**

Oral diseases are a major global health challenge, posing health and economic burdens that have profound impacts on the quality of life, disproportionately affecting marginalized populations such as tribal communities. Among scheduled tribes in India, the Irula community belongs to one of the tribes most vulnerable by poor access to health care and education. The elders in the population have increased incidence and prevalence rates of tooth morbidities-an increased incidence of caries, non-carious lesions, and periodontitis-related conditions that affect oral health related quality of life (OHQOL) dimensions.

**Objectives:**

This study aims to assess tooth morbidity and tooth loss among older Irula community members, identify risk factors, and evaluate how oral and physical comorbidities have been associated with OHRQoL.

**Methods:**

A cross-sectional study was conducted on elderly persons aged 60 years and above in Thiruvallur district, Tamil Nadu, India. A multi-stage random sampling technique was employed in the study. Data were collected by semi-structured questionnaires covering demographics, medical history, oral health practices, and quality of life by clinical dental examination. The tools used included the International Caries Detection and Assessment System (ICDAS) the Smith and Knight Tooth wear index for non-carious lesions, and the Community Periodontal Index for Treatment Needs (CPITN) index for periodontal disease Furthermore, quality of life assessment was carried out utilizing the Geriatric Oral Health Assessment Index (GOHAI). Subsequently, Descriptive and multivariate logistic regression analyses were performed to determine the predictors of OHRQoL.

**Results:**

The prevalence of carious lesions was 38.5%, non-carious lesions 70%, periodontitis 70.8%, and tooth loss 53.6%. Only 2.3% of participants had restorative dental treatments. Multivariate analysis reveals that illiteracy (AOR = 0.163, *p* = 0.003), arthritis (AOR = 0.340, *p* = 0.001), carious lesions (AOR = 1.402, *p* = 0.031), periodontal disease (AOR = 1.663, *p* = 0.002), and tooth loss (AOR = 2.744, *p* = 0.001) affected OHRQoL significantly.

**Conclusion:**

The results regarding the elderly community of Irula point towards the severe oral health disparities observed among them; thus, this raises an urgent need to develop a public health intervention for this eminent existing fact. Removing education and socioeconomic barriers, improving access to dental health care, and advocating culturally appropriate preventive programs could increase OHRQoL. Longitudinal study and policy-driven approaches should be carried out in future studies for sustainable health equity of the tribal population.

## Introduction

Oral diseases are a major global health challenge, posing health and economic burdens that have profound impacts on the quality of life [1]. More than 3.5 billion cases are reported globally, most of which can be avoided [2]. Their prevalence and severity, however, are compounded by the increase in chronic diseases, particularly in aging populations [3]. Quality of life is defined as a multidimensional variable that includes physical, mental, and social well-being by WHO. This perception of situation is influenced by multiple factors, such as physical environment, job satisfaction, education, intellectual and social satisfaction and wider factors such as freedom, justice and freedom from oppression [4].

Tribal communities are an important portion of indigenous populations, making up 8.6% of India’s population. Many of these live in central and western areas, often in remote mountainous regions with little or no access to technology, education, and economic opportunities, and are therefore, some of the most marginalized [5]. Scheduled Tribes (ST), comprising 1.1% of the population in Tamil Nadu, includes 36 listed groups. Of these groups, the Kattunayakan, Kotas, Irulas, Paniyas, Kurumbas, and Todas are defined as Particularly Vulnerable Tribal Groups (PVTG) by the Indian Government [6].

The name for the Irula community comes from the Tamil word ‘Irul’ which means ‘darkness’ and they mainly inhabit in the districts of Thiruvallur, Kancheepuram, and Tiruvannamalai [7]. However, while it may be the case with other tribes, there is a lack of research regarding this communities oral health [8–10]. Therefore, addressing the oral health disparities of Irula community becomes imperative because the Irula community has different health needs [11].

Tooth morbidity comprising dental decay, non-carious lesions and periodontal disease remains a worldwide problem, common up to 45% of the population in the last 30 years [12]. Caries affecting teeth and periodontitis are dominant among the cause of losing teeth which means high costs on rehabilitation and losses to the social wellbeing of individuals as well [13].

This cross-sectional study seeks to evaluate the prevalence of tooth morbidity and tooth loss while identifying risk factors for conditions such as caries, non-carious lesions, and periodontitis within the Irula tribes. The study focuses specifically on elderly individuals, as they represent a particularly vulnerable subgroup in the community. Older adults face heightened susceptibility to oral health issues due to limited access to healthcare, cultural practices, socioeconomic challenges, and insufficient awareness about oral hygiene. By concentrating on this age group, the study aims to shed light on their unique oral health challenges and inform targeted interventions for this underserved population.

## Methods

### Study design and setting

A cross-sectional study design was selected for this research as it provides an effective method for capturing the current state of oral health and associated factors within the study population at a specific point in time. This approach is particularly useful for assessing the prevalence of conditions such as tooth morbidity and Qol while also identifying potential risk factors related to demographic, behavioral, and socioeconomic variables. The study was carried out among Irula tribes in Thiruvallur district, Tamil Nadu, India. Participants above the age of 60 years and participants who have been residing for minimum duration of 6 months were included. Chronically ill patients with restricted movements and subjects who do not give consent to the examination were excluded from the study.

### Sample size

Using a prevalence rate of 50.1% from a study by Shah et al. [14], the sample size was calculated with a confidence interval of 98%, a 5% error term, a design effect of 2%, and a 10% non-response rate, resulting in an overall sample of 881 individuals.

### Sampling technique

The study utilized a multi-stage sampling method to select a representative sample of elderly individuals from the western part of Chennai, ensuring randomness at each stage of participant selection. The process involved several stages: first Stage (District Selection): The study began by selecting Thiruvallur district, which consists of 9 taluks. Two taluks were randomly chosen using a lottery method. To ensure balanced representation, the selection process considered the number of villages in each taluk, so that the random selection reflected the geographical distribution of villages. Second Stage (Taluk Selection): From the 9 taluks, two were selected: Pallipattu and Tiruttani, which together have a total of 21,366 Irula households. Randomness was maintained by drawing the taluks without bias, ensuring an equal chance of selection for each taluk. Third Stage (Household Selection): In the final stage, list of household was obtained from Block development officer among them one eligible elderly individual was randomly selected. This process continued until the required sample size was met, ensuring that the selection of participants was unbiased and that each elderly individual had an equal opportunity of being included in the study.

The dependent variable is including quality of life, while the independent variables include socio-economic factors, Tooth morbidity (caries, Non carious lesions, periodontitis and tooth loss), and physical co-morbidities.

### Study procedure

A single trained examiner conducted the interviews for the study. The research objectives were explained to the participants, who then provided signed informed consent. The questionnaire, administered in Tamil, covered a range of topics including demographic details, educational background, family information, economic status, personal habits, general health and medical conditions, oral health and dental issues, as well as awareness of dental care.

### Study tool

A semi-structured questionnaire was designed based on a comprehensive review of existing literature, scientific publications, and research studies. It was adapted to incorporate the International Caries Detection and Assessment System (ICDAS) for caries detection [15], the Smith and Knight Tooth Wear Index for evaluating non-carious lesions [16], and the Community Periodontal Index for Treatment Needs (CPITN) for assessing periodontal disease [17]. Furthermore, the Geriatric Oral Health Assessment Index (GOHAI) was utilized to evaluate oral health-related quality of life [18].The questionnaire was converted into a digital format and uploaded onto portable computing devices. The study employed various instruments, including curved probes, straight probes, mouth mirrors, WHO probes (CPITN), cotton rolls, chip blowers, and explorers for dental examination purposes. Pilot testing was done and the study shows high internal consistency with cronbach’s alpha values above 0.75 that is **0.93**. Face validity and content validity questionnaire was established through expert reviews, dental professionals, public health experts, pilot testing with the target population, and feedback from laypersons, ensuring clarity, relevance, and comprehensiveness.

### Data analysis

Data from this study were entered into Microsoft excel, cleaned, and saved for further analysis. Analysis was done using SPSS Statistics V.23 (IBM SPSS Statistics). The aim of the analysis was to understand the relationships between socio-demographic characteristics, oral health conditions, and physical comorbidities with their effects on OHRQoL. Descriptive statistics were employed for summarizing socio-demographic characteristics and oral health conditions as well as for the prevalence of oral diseases: caries, non-carious lesions, periodontitis, and tooth loss. Frequencies and percentages were computed for socio-demographic variables including age, gender, education, marital status, employment, and income. GOHAI was utilized to evaluate QoL. Higher scores represented a better quality of oral health-related life. Bivariate analysis, which consisted of Chi-square test was conducted to find associations between independent variables, which included socio-demographic factors, oral health conditions, and physical comorbidities, and OHRQoL. Logistic regression was used to examine associations between oral health conditions, physical comorbidities and OHRQoL, adjusting for potential confounding related to socioeconomic factors. The GOHAI scores were used to classify individuals as having poor or good quality of life based on a median score cut-off of 2 [19]. Adjusted odds ratio was further calculated with its respective 95% CI in the estimate of strength of association with values *p* < 0.05 for statistical significance, then proceeded to check up model’s goodness of fit which ensures appropriateness or fitting of the logistic regression model.

## Results

The study sample was primarily composed of individuals in the 60–64 age group, making up 44.8% of the population, followed by those in the 65–69 age group at 30.6%. A notable majority of the participants were female (76.4%), while males represented 23.6%. The majority identified as Hindu (94.1%), with a smaller proportion identifying as Christian (5.9%). In terms of marital status, most participants were married (72.5%), while 27.5% were widowed. When considering educational background, a large proportion of the sample was illiterate (61.9%), with 11.1% having received primary education and 10.2% having completed high school. The majority of participants lived in nuclear families (87.6%), while a smaller group resided in joint family setups (12.4%). Employment-wise, most individuals worked as agricultural laborers (60.0%), followed by housewives (20.4%) and those who were either unemployed or retired (6.7%). Housing conditions revealed that 62.4% lived in semi-pucca houses, with 33.9% residing in katcha houses. Income distribution showed that a significant portion earned less than Rs. 5,000 (54.9%), with 39.3% earning between Rs. 5,001 and Rs. 10,000. A summary of the socio-demographic characteristics can be found in Table 1.

**Table 1:** Socio demographic variables of the participants (*n* = 881)

Category	Subcategory	Percent	Frequency
Age	60–64 years	44.80%	395
	65–69 years	30.60%	270
	70–74 years	10.80%	95
	75–79 years	9.20%	81
	80–84 years	3.10%	27
	85–89 years	1.50%	13
Gender	Female	76.40%	673
	Male	23.60%	208
Religion	Christian	5.90%	52
	Hindu	94.10%	829
Marital Status	Married	72.50%	639
	Widow	27.50%	242
Educational Qualifications	Primary school and below	73.9%	651
	Middle School and Above	26.1%	230
Family Type	Joint Family	12.40%	109
	Nuclear Family	87.60%	772
Type of Occupation	Agricultural Labourers	586	66.5
	House Wife	180	20.4
	Private	15	1.7
	Small Business	30	3.4
	Retired/Currently Unemployed	59	6.7
	Others	11	1.2
Type of House	Katcha	299	33.9
	Pucca	32	3.6
	Semi Pucca	550	62.4
Monthly Income	Less than 5000	484	54.9
	5001–10,000	346	39.3
	10,001–20,000	51	5.8

**Fig. 1:**
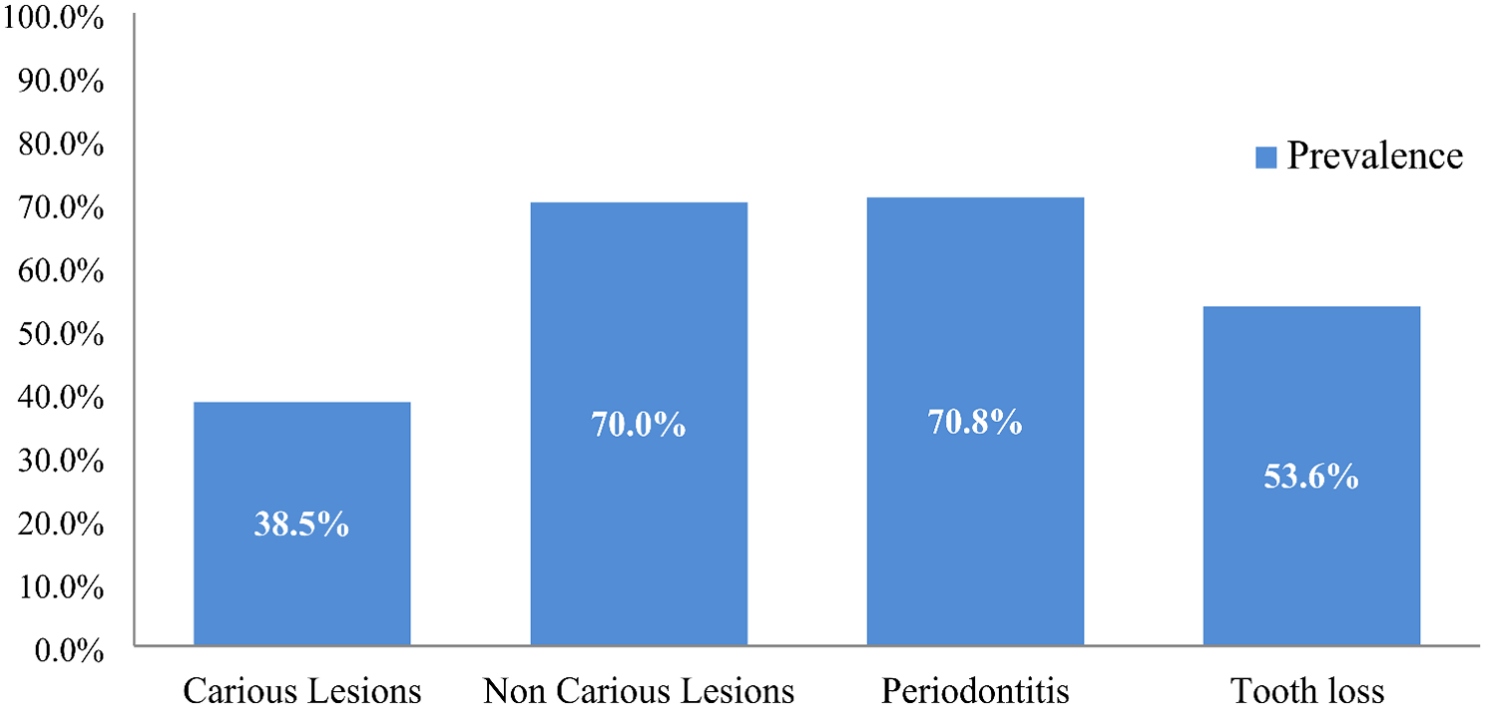
Prevalence of tooth morbidity

The prevalence of carious lesions was observed to be 38.5% (95% CI: 35.3 − 41.7%), indicating a significant portion of the population affected by dental decay. Non-carious lesions were notably more prevalent, affecting 70% of individuals (95% CI: 67 − 73.1%), reflecting a widespread occurrence of dental conditions beyond cavities. Periodontitis was prevalent in 70.8% of the population (95% CI: 68 − 73.8%), highlighting the high burden of gum disease. Additionally, tooth loss was reported in 53.6% of participants (95% CI: 50.3 − 56.9%), further emphasizing the extent of dental health challenges within this population (Fig. 1).

Of the participants 21.9% of them reported they are frequently limiting the amount of food they consume due to issues with their teeth or dentures. Similarly, 20.7% reported always experiencing trouble biting or chewing different types of food, while 21.0% stated they are always able to swallow comfortably. Teeth or dentures prevent 19.1% of respondents from speaking as desired only 19.8% indicated they are always able to eat without discomfort. Social limitations due to oral health issues were reported by 18.0% of participants who frequently limit contact with others because of their teeth or dentures. Positive self-perception is seen in only 19.4% of participants who are always happy with the look of their teeth and gums or dentures. The use of medication to relieve pain or discomfort around the mouth is always necessary for 18.0% of respondents. 18.3% of the participants always worry about their oral health problems and 18.6% always feel nervous or self-conscious because of these issues. Similarly, 18.3% always feel uncomfortable eating in front of others due to teeth or dentures. Finally, sensitivity to hot, cold or sweet foods is experienced always by 21.6% of participants. For the quality of life (QOL) assessment, the responses of the 881 participants are summed across the 12 statements to yield an overall score ranging from 0 to 36 median score of 2 is used as the reference point [19], which serves as a threshold to differentiate between poor and good quality of life. Scores below or equal to 2 indicate poor quality of life, while scores above 2 suggest a good quality of life. Oral health related quality of life (QoL) was good for 47% of the population in question, while for 53%, it was poor.

Table 2 describes the factors influencing Oral Health-Related Quality of Life (OHRQoL). Age and Educational attainment emerged as a significant determinant of OHRQoL, with individuals lacking primary education and below (AOR = 1.507, 95% CI: 1.051–2.161, *p* = 0.026), exhibiting poorer OHRQoL compared to middle school and above. Systemic conditions such as diabetes (AOR = 0.727, 95% CI: 0.504–1.049, *p* = 0.088) were not independently associated with OHRQoL. In contrast, specific oral health conditions demonstrated significant associations with impaired OHRQoL, including carious lesions (AOR = 1.369, 95% CI: 1.002–1.870, *p* = 0.049), periodontitis (AOR = 1.686, 95% CI: 1.213–2.343, *p* = 0.002), and tooth loss (AOR = 2.752, 95% CI: 2.030–3.730, *p* < 0.001), each contributing to substantial declines in quality of life.

**Table 2:** Multivariate logistic regression analysis of risk factors, oral and physical comorbidities affecting quality of life

Variables		Unadjusted Odds ratio(95%CI)	*p* value	Adjusted Odds ratio(95%CI)	*p* value (< 0.05)
Age	60–65	Reference	Reference	Reference	Reference
	More than 65	0.639 (0.488–0.837)	0.001*	1.685 (1.241–2.287)	0.001*
Gender	Female	Reference	Reference	Reference	Reference
	Male	1.362 (0.998–1.861)	0.05	0.817 (0.577–1.156)	0.254
Education	Middle School and above	Reference	Reference	Reference	Reference
	Primary school and below	0.445 (0.325–0.611)	0.001*	1.507 (1.051–2.161)	0.026*
Diabetes	No	Reference	Reference	Reference	Reference
	Yes	1.851 (1.328–2.579)	0.002*	0.727 (0.504–1.049)	0.088
Carious Lesions	No	Reference	Reference	Reference	Reference
	Yes	0.565 (0.429–0.745)	0.001*	1.369 (1.002–1.870)	0.049*
Non Carious Lesions	No	Reference	Reference	Reference	Reference
	Yes	5.614 (3.992–7.896)	0.001*	0.202 (0.141–0.292)	0.001*
Periodontitis	No	Reference	Reference	Reference	Reference
	Yes	0.560 (0.417–0.751)	0.001*	1.686 (1.213–2.343)	0.002*
Tooth Loss	No	Reference	Reference	Reference	Reference
	Yes	0.376 (0.286–0.495)	0.001*	2.752 (2.030–3.730)	0.001*

*, Statistical significance at *p* < 0.05;

## Discussion

Oral diseases are often considered as “neglected epidemic”. They significantly impact overall health and place a heavy burden on individuals and healthcare systems [20].Dental caries, often associated with modernization and lifestyle changes, present a significant threat to geographically remote and culturally traditional populations [21]. Factors such as limited access to dental care services, passive approaches to oral health, and low levels of awareness, financial hardships, and widespread illiteracy amplify the risk of dental caries in these communities [22]. Nonetheless, indigenous populations experience significant health inequalities, which are largely caused by their lower socioeconomic level in comparison to the overall population, despite several government measures targeted at improving their health outcomes [23]. In a recent study conducted by World health organization in collaboration with the Government of India to find the health problems of elderly population (aged 60 years and above), it was found that a large proportion i.e., 32.6% had dental problems apart from other medical problems like diabetes, hypertension and ischemic heart disease [24]. A review article dome among the Indian population shows 73.2% of Periodontitis was found among elderly population [25] which is similar to our study results. A systematic review findings shows caries in older adults is still a major public health issue, even in developed countries [12]. A study reported the caries risk in older adults increases by 60% as there is low resting pH and low stimulated salivary flow rate. Alongside dental caries, chronic periodontitis is the leading cause of tooth loss for adults globally [26]. In the Global Burden of Disease Study, untreated caries was the most prevalent, affecting 3.1 billion people (44%) worldwide, with a major impact on quality of life and high costs for individuals, families and society [27]. Worldwide, the prevalence of dental caries among adults is high as the disease affects nearly 100% of the population in the majority of countries [28]. In several industrialized countries, older people have often had their teeth extracted early in life because of pain or discomfort, leading to reduced quality of life [29]. Tooth wear prevalence is high and increasing and has important consequences on the patient’s quality of life [30]. This research is the first of its kind to comprehensively examine all aspects of tooth morbidity among older adults within the Irula tribal population, along with the associated risk factors. Education, caries, periodontitis and tooth loss are the important variables that affect OHRQoL. Oral health deteriorates with aging, resulting in tooth loss, attachment loss, and poor oral hygiene [31]. Despite the fact that these diseases are frequently treatable or reversible, many elderly people do not seek appropriate care. This might be because people above the age of 60 were not exposed to preventative dental techniques as children and are hence less likely to adopt them [32]. Furthermore, some people feel that tooth loss is an unavoidable component of becoming older and cannot be prevented. Others have adopted a lower standard of oral health and only seek care in crises [33]. This study revealed that senior respondents reported more physical functional issues and less psychological problems, most likely due to a high prevalence of untreated dental abnormalities.

### Limitations

The study has few limitations that should be acknowledged. First, the cross-sectional design restricts the ability to establish causality. Additionally, recall bias is a concern, as participants may inaccurately remember or report past oral or health issues. However, efforts were made to minimize this bias by using probing questions to clarify responses, and participants were prompted to recall information from within past one-month period to enhance accuracy. However Cross-sectional studies on tribal populations often face limited generalizability due to unique cultural contexts. Findings may not apply across diverse tribal groups or changing circumstances.

### Recommendations

Oral health disparities in tribal communities, especially in the elderly Irula community, can be addressed through interventions such as awareness programs should be initiated to educate about oral hygiene and its importance. Improvement of accessibility through mobile dental clinics and free or subsidised dental care can reduce the barriers of treatment, especially in the remote areas. Oral health screening could be integrated into routine health check-ups. Policy interventions should support health equity by integrating oral health into primary healthcare policies, expanding dental coverage under health insurance, and securing specific funding for tribal regions.

## Conclusion

This study found that there is an oral health disparity within the Irula tribal population, particularly among elderly individuals who face unique vulnerabilities. The high prevalence of caries, non-carious lesions, periodontitis, and tooth loss underscores the pressing oral health challenges within this marginalized group. Factors such as limited education, socioeconomic hardships, and chronic conditions like arthritis further exacerbate the impact on their OHRQoL. These findings emphasize the importance of targeted public health interventions, including education programs, improved access to dental care, and culturally sensitive preventive strategies. Future research should explore longitudinal studies and implement policy-driven approaches to provide sustainable oral health solutions for tribal populations, fostering health equity and social inclusion.

## Data Availability

The data that support the findings of this study are available on request from the corresponding author (alexjosephdr@gmail.com).
